# The GLP-1 receptor agonists exendin-4 and liraglutide alleviate oxidative stress and cognitive and micturition deficits induced by middle cerebral artery occlusion in diabetic mice

**DOI:** 10.1186/s12868-016-0272-9

**Published:** 2016-06-13

**Authors:** Ping-Chia Li, Li-Fen Liu, Ming-Jia Jou, Hao-Kuang Wang

**Affiliations:** School of Medicine for International Students, I-Shou University (Yanchao Campus), Kaohsiung, Taiwan; Department of Occupational Therapy, College of Medicine, I-Shou University (Yanchao Campus), Kaohsiung, Taiwan; Department of Neurosurgery, E-Da Hospital, I-Shou University, Kaohsiung, Taiwan

**Keywords:** Cerebral microcirculation, GLP-1 agonist, Middle cerebral artery occlusion, Oxidative stress, Pelvic nerve, Voiding function

## Abstract

**Background:**

Glucagon-like peptide 1 (GLP-1) analogs protect a variety of cell types against oxidative damage and vascular and neuronal injury via binding to GLP-1 receptors. This study aimed to investigate the effects of the GLP-1 analogs exendin-4 and liraglutide on cerebral blood flow, reactive oxygen species production, expression of oxidative stress-related proteins, cognition, and pelvic sympathetic nerve-mediated bladder contraction after middle cerebral artery occlusion (MCAO) injury in the db/db mouse model of diabetes.

**Results:**

Sixty minutes of MCAO increased blood and brain reactive oxygen species counts in male db/db mice, as revealed by dihydroethidium staining. MCAO also increased nuclear factor-κB and intercellular adhesion molecule-1 expression and decreased cerebral microcirculation. These effects were attenuated by treatment with exendin-4 or liraglutide. MCAO did not affect basal levels of phosphorylated Akt (p-Akt) or endothelial nitric oxide synthase (p-eNOS); however, exendin-4 and liraglutide treatments significantly enhanced p-Akt and p-eNOS levels, indicating activation of the p-Akt/p-eNOS signaling pathway. MCAO-induced motor and cognitive deficits and micturition dysfunction, indicated by reduced pelvic nerve-mediated voiding contractions and increased nonvoiding contractions, were also partially attenuated by exendin-4 treatment.

**Conclusions:**

The above data indicate that treatment with GLP-1 agonists exerts protective effects against oxidative, inflammatory, and apoptotic damage in brain areas that control parasympathetic/pelvic nerve-mediated voiding contractions and cognitive and motor behaviors in a diabetic mouse model.

## Background

Diabetes mellitus is a major independent risk factor for stroke occurrence; patients with type 2 diabetes have twice the risk of stroke compared to the general population [1] and are at markedly increased risk of death due to cerebrovascular disease [2, 3]. The precise mechanism for diabetes-induced cerebral and vascular damage may involve a causal relationship between hyperglycemia-evoked oxidative stress and inflammation [4–7]. Type 2 diabetes can cause oxidative stress, leading to vascular complications and stroke [8–11]. Acute hemispheric stroke may, in turn, result in urinary incontinence. Bladder dysfunction has been reported in patients suffering from stroke [12]. Additionally, rats subjected to middle cerebral artery occlusion (MCAO), which disrupts the cholinergic pathway between the frontal cortex and the nucleus basalis [13], show increased voiding frequency, inefficient voiding [14], and decreased bladder capacity [15]. Treating MCAO injury by intracerebrovascular administration of the acetylcholinesterase inhibitor donepezil increases bladder capacity [15].

Glucagon-like peptide-1 (GLP-1) is a gut hormone secreted by the small intestine that exerts a glucose-dependent stimulatory effect on insulin release by binding to GLP-1 receptors (GLP-1Rs) on pancreatic β-cells [16, 17]. GLP-1 also stimulates β-cell proliferation [18] and enhances β-cell differentiation [19]. GLP-1 and its longer-acting analogs exendin-4 (Ex-4) and liraglutide (Lir) protect a variety of cell types against oxidative injury and are widely used in therapeutic contexts. These agents exert protective effects in experimental models of dilated cardiomyopathy, arteriovenous failure [20], myocardial infarction [21], and global stroke [3]. GLP-1Rs are highly expressed throughout the brain [22, 23], and GLP-1 and GLP-1R agonists such as Ex-4 completely protect against glutamate–induced neuronal death [24] and stroke injury [3]. Ex-4 has been shown to attenuate neointimal hyperplasia after vascular injury [25] and prevent lipotoxicity-induced apoptosis in murine pancreatic β-cells through activation of Akt and inhibition of the mitochondrial pathway [26]. Ex-4 also reduces damage to vascular endothelial cells by high levels of glucose and tumor necrosis factor-α by inhibiting p38 mitogen-activated protein kinase expression and the nuclear translocation of nuclear factor kappa B (NF-κB) [27]. Lir protects against oxidative stress and diabetic nephropathy via a PKA-mediated inhibition of renal nicotinamide adenine dinucleotide phosphate (NADPH) oxidase [28].

Given the prevalence of stroke in diabetic patients, therapeutic strategies for reducing stroke-induced motor and cognition deficits and bladder dysfunction in these patients after acute stroke are of interest. Based on the previously established protective effects of GLP-1 analogs against oxidative stress and cell death, we investigated the effects of Ex-4 and Lir on cerebral microcirculation, oxidative stress-related protein expression, cognitive and motor behaviors, and parasympathetic nerve-mediated micturition function after MCAO in the db/db mouse model of diabetes.

## Results

### Ex-4 and Lir partially alleviate MCAO-induced reductions in cerebral microcirculation

**Fig. 1:**
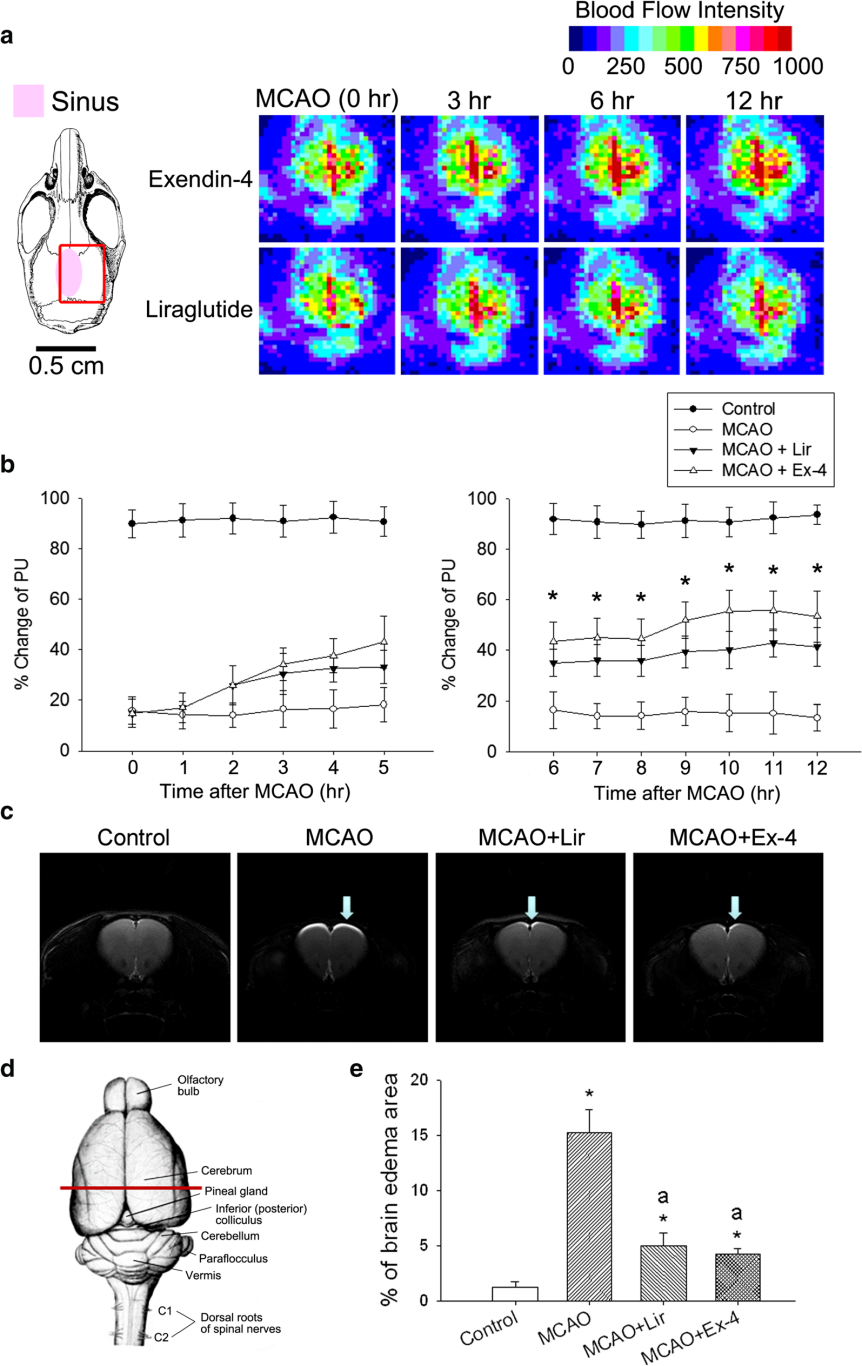
Effects of exendin-4 and liraglutide on cerebral microcirculation after injury in db/db mice. **a** Schematic diagram showing the area of microcirculation measurement. **b** Representative full field laser perfusion images showing cerebral microcirculation in mice treated with exendin-4 (Ex-4) or liraglutide (Lir) following middle cerebral artery occlusion (MCAO) injury. Percentage change in perfusion units (PU), presented as mean ± standard error of the mean (SEM; n = 6 per condition). Groups were compared using two-way analysis of variance (ANOVA) with a post hoc Bonferroni adjustment for pairwise comparisons. *P < 0.05 compared to the MCAO group. **c** The T2-weight magnetic resonance imaging series revealed that after MCAO 12 h group marked brain edema (hight signal intensity area) had a significantly higher percentage in the *red line* section area **d** in comparison with control group. **e** After Exendin-4 and Liraglutide treatment decrease the brain edema percentage more significantly than the group of MCAO. *P < 0.05 compared to the control group, ^a^ *P* < 0.05 compared to the MCAO group

Figure 1 shows changes in cerebral microcirculation in the right hemisphere of db/db mice following MCAO injury. Cerebral microcirculation was significantly reduced throughout the 12 h following MCAO compared to that in the control group (Fig. 1a, b). Within 6–12 h after MCAO, mice treated with Ex-4 or Lir showed significantly increased microcirculation compared to the MCAO group. There was no significant difference in the percentage change in perfusion units (PU) between mice treated with Ex-4 and Lir. Brain edema was not observed via T-2-weighted MRI in control group. Marked forebrain edema in the section area 12 h after MCAO group was significantly increased in the MCAO group compared to that in the control group (Fig. 1c, d). Between mice treated with Ex-4 or Lir significantly decreased edema area compared to MCAO group (Fig. 1c, e).

### Ex-4 reduces MCAO-induced expression of oxidative stress and apoptosis markers

**Fig. 2:**
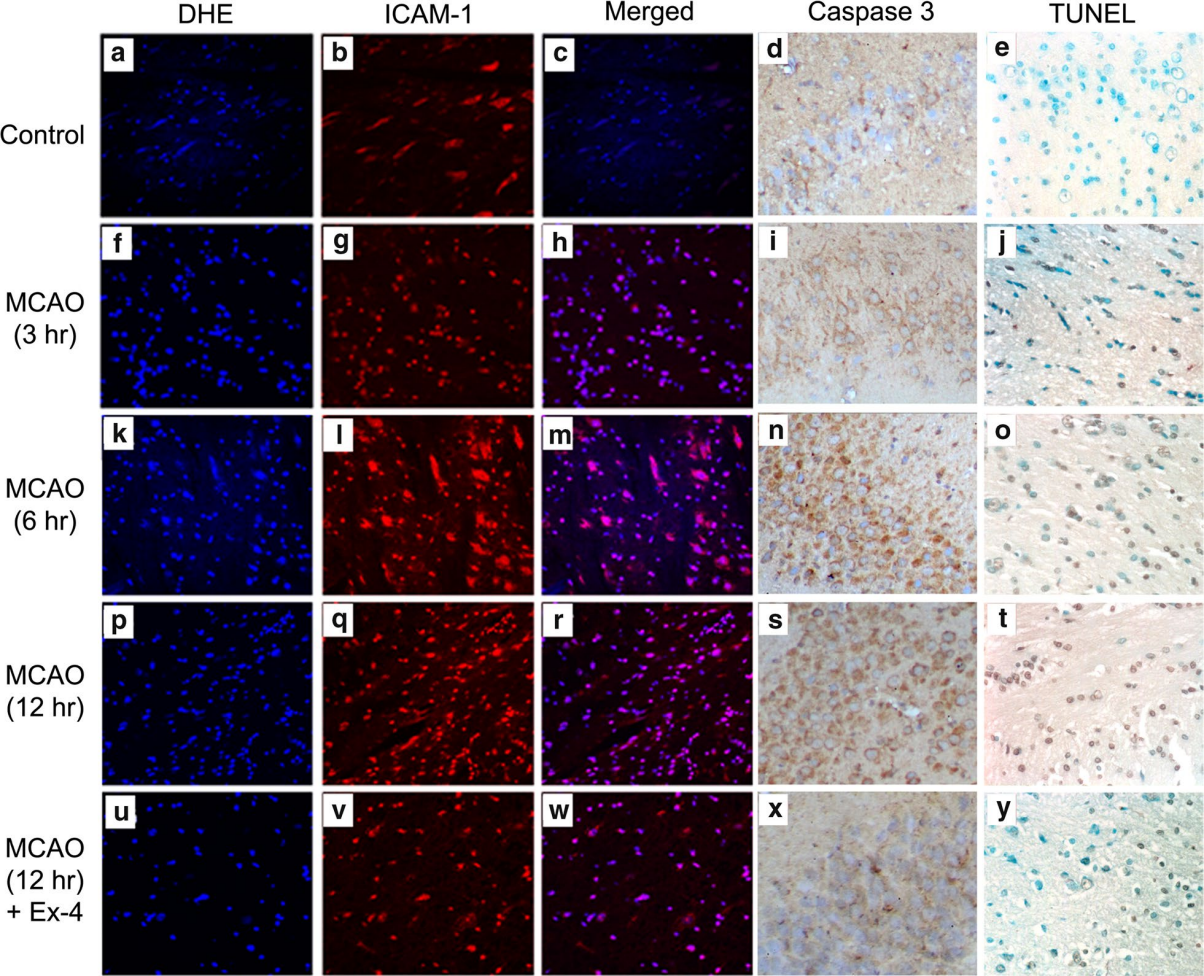
Effects of exendin-4 on MCAO-induced markers of oxidative stress, inflammation, and apoptosis. Shown are dihydroethidium (DHE) labeling of reactive oxygen species (ROS; **a**, **f**, **k**, **p**, **u**), intercellular adhesion molecule-1 (ICAM-1; **b**, **g**, **l**, **q**, **v**), DHE and ICAM-1 merged (**c, h, m, r, w**) caspase 3 (**d**, **i**, **n**, **s**, **x**, indicated by *brown color*) and terminal deoxynucleotidyl transferase dUTP nick end labeling (TUNEL; **e**, **j**, **o**, **t**, **y**, indicated by *brown color*) at 3, 6, and 12 h after MCAO injury in db/db mice. MCAO markedly increased DHE, ICAM-1, Caspase 3 and TUNEL labeling; these effects were offset by Ex-4 treatment (magnification DHE, ICAM-1 400; and magnification Caspase3, TUNEL 200)

As shown in Fig. 2, MCAO markedly increased staining for the ROS marker dihydroethidium (DHE) and the inflammatory marker intercellular adhesion molecule-1 (ICAM-1) in the right brainstem of db/db mice. Mice subjected to MCAO also showed increased caspase 3 expression and terminal deoxynucleotidyl transferase-mediated dUTP nick end labeling (TUNEL), which are markers of apoptosis. Staining for DHE, ICAM-1, caspase 3 and TUNEL was greater at 12 h than at 3 or 6 h after MCAO. These MCAO-induced increases in oxidative stress and apoptotic markers were markedly decreased by Ex-4 treatment (Fig. 2u–y).

**Fig. 3:**
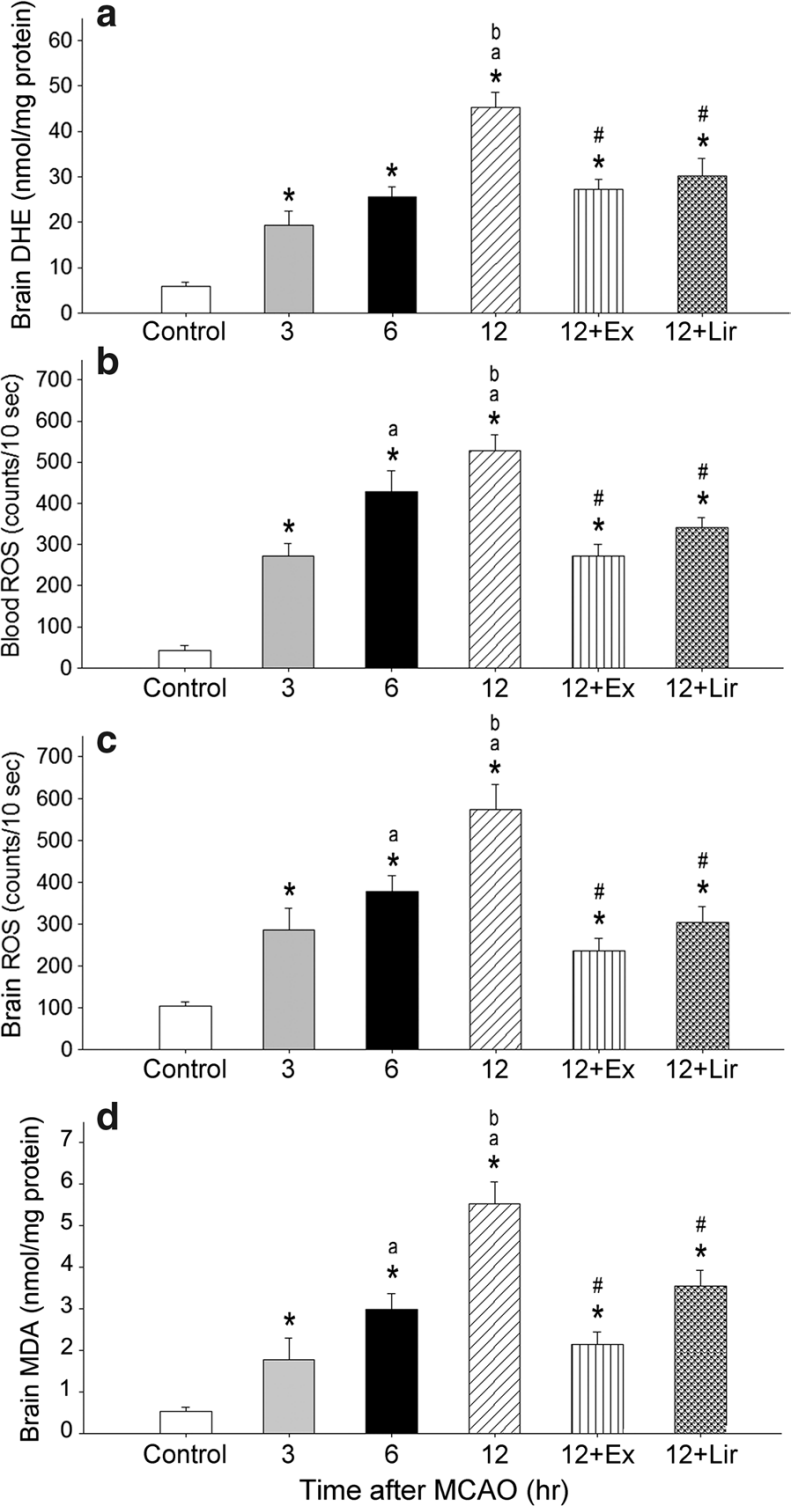
Effects of exendin-4 and liraglutide on MCAO-induced ROS production. Shown are DHE (**a**), blood ROS (**b**), brain ROS (**c**) and brain malondialdehyde (MDA) content (**d**) at 3, 6, and 12 h after MCAO injury in db/db mice. MCAO significantly increased DHE expression, blood and brain ROS levels, and brain MDA content, whereas exendin (Ex) and Lir partially occluded these effects. Data are presented as mean ± SEM (n = 6 per condition). Groups were compared using two-way ANOVA with a post hoc Bonferroni adjustment for pairwise comparisons. *P < 0.05 compared to controls; ^a^ *P* < 0.05 compared to 3 h after MCAO; ^b^ *P* < 0.05 compared to 6 h after MCAO; ^#^ *P* < 0.05 compared to 12 h after MCAO

MCAO also significantly increased the levels of brain DHE labeling (Fig. 3a), blood and brain reactive oxygen species (ROS) (Fig. 3b, c), and brain malondialdehyde (MDA) (Fig. 3d), a by-product of lipid peroxidation that indicates oxidative stress. Levels of these markers were higher at 12 h than at 3 or 6 h after injury in the MCAO group but were significantly reduced by Ex-4 and Lir treatments (*P* < 0.05). Ex-4 and Lir displayed similar effects in reducing brain and blood markers of oxidative stress.

### Ex-4 and Lir inhibit MCAO-induced inflammatory protein expression

**Fig. 4:**
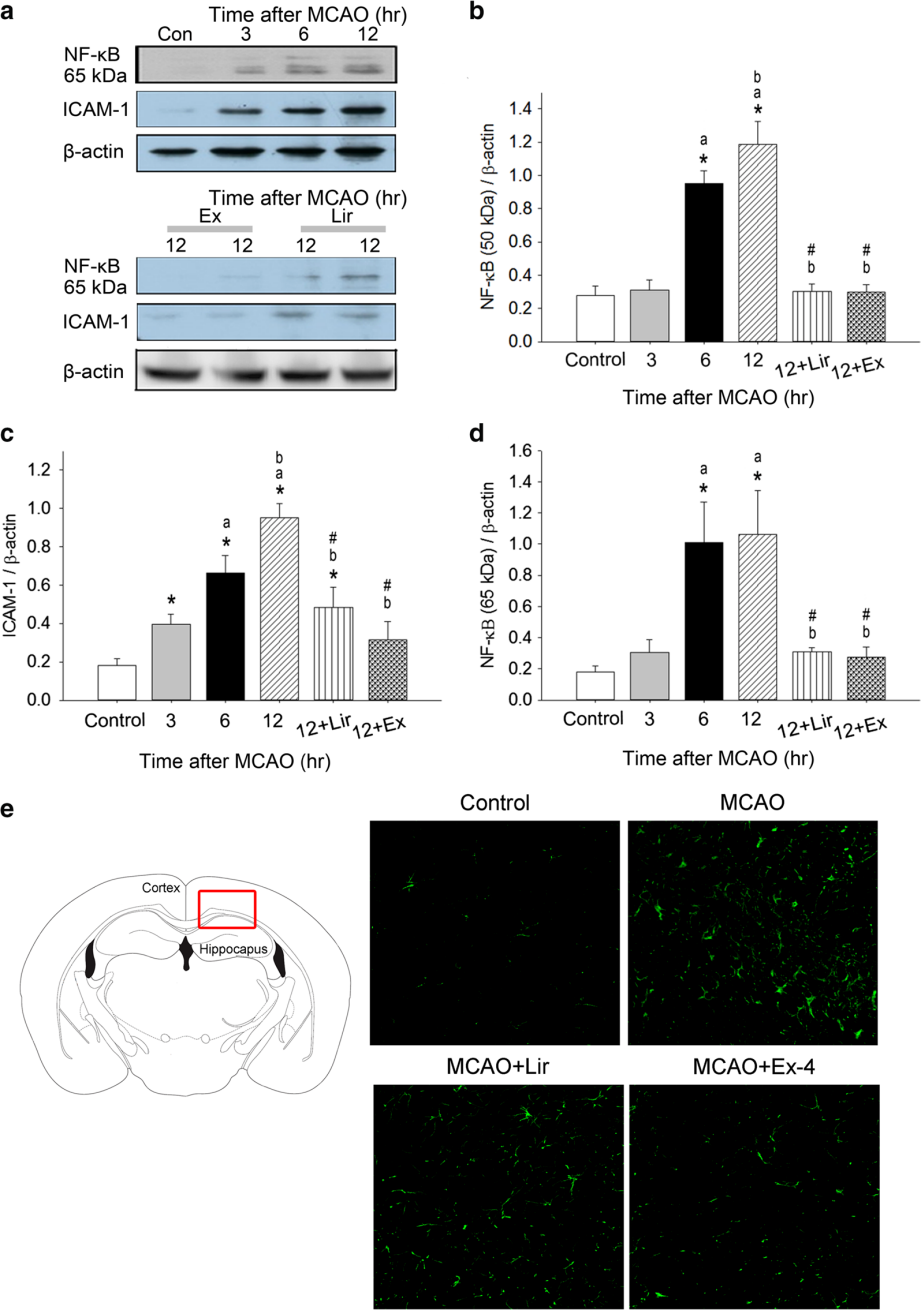
Effects of exendin-4 and liraglutide on MCAO-induced inflammatory protein expression. **a** Typical western blots of nuclear factor-κB (NF-κB) p65, and ICAM-1 in cortical extracts at 3, 6, or 12 h after MCAO. Densitometry of **b** NF-κB50, **c** ICAM-1, and **d** NF-κB p65 bands normalized to β-actin. Data are presented as mean ± SEM (n = 6 per condition). **e** The expression of NF-κB (green fluorescence) was markedly enhanced in the cerebral cortical area 12 h after MCAO in comparison with the matched-time control mice. The increased NF-κB fluorescence was efficiently attenuated in the Lir- or Ex-4-treatment group 12 h after MCAO. The NF-κB fluorescence seems to be expressed in both neuronal and glial cells 12 h after MCAO (Magnification 400×). Groups were compared using two-way ANOVA with a post hoc Bonferroni adjustment for pairwise comparisons. *P < 0.05 compared to controls; ^a^ *P* < 0.05 compared to 3 h after MCAO; ^b^ *P* < 0.05 compared to 6 h after MCAO; ^#^ *P* < 0.05 compared to 12 h after MCAO

Expression of the inflammation-related proteins ICAM-1, NF-κB p50, and NF-κB p65 in db/db mice following MCAO, as measured by western blotting [29], is shown in Fig. 4. ICAM-1 expression was significantly higher greater at 12 h than at 3 h after MCAO and than in controls; this increase was significantly suppressed by Ex-4 and Lir treatments. Ex-4 decreased ICAM-1 expression to a slightly greater extent than did Lir, although this difference was not significant. NF-κB p50 and p65 expression in the right hemisphere of cortical homogenate was significantly increased 6 and 12 h after MCAO compared to controls (Fig. 4b, d). Ex-4 and Lir exerted similar effects on MCAO-induced increases in cortical NF-κB p50 and p65 expression. These increases were significantly inhibited 12 h after MCAO by Ex-4 and Lir treatments. As shown in Fig. 4e, the green NF-κB fluorescence was greatly increased in the section of cerebral cortical area 12 h after MCAO in comparison with the matched-time control mice. The enhanced NF-κB fluorescence was effectively depressed in the Lir- or Ex-4-treatment group 12 h after MCAO. According to cortical cellular morphology, NF-κB fluorescence seems to be expressed in both neuronal and glial cells 12 h after MCAO.

### Ex-4 and Lir repress MCAO-induced expression of apoptotic markers

**Fig. 5:**
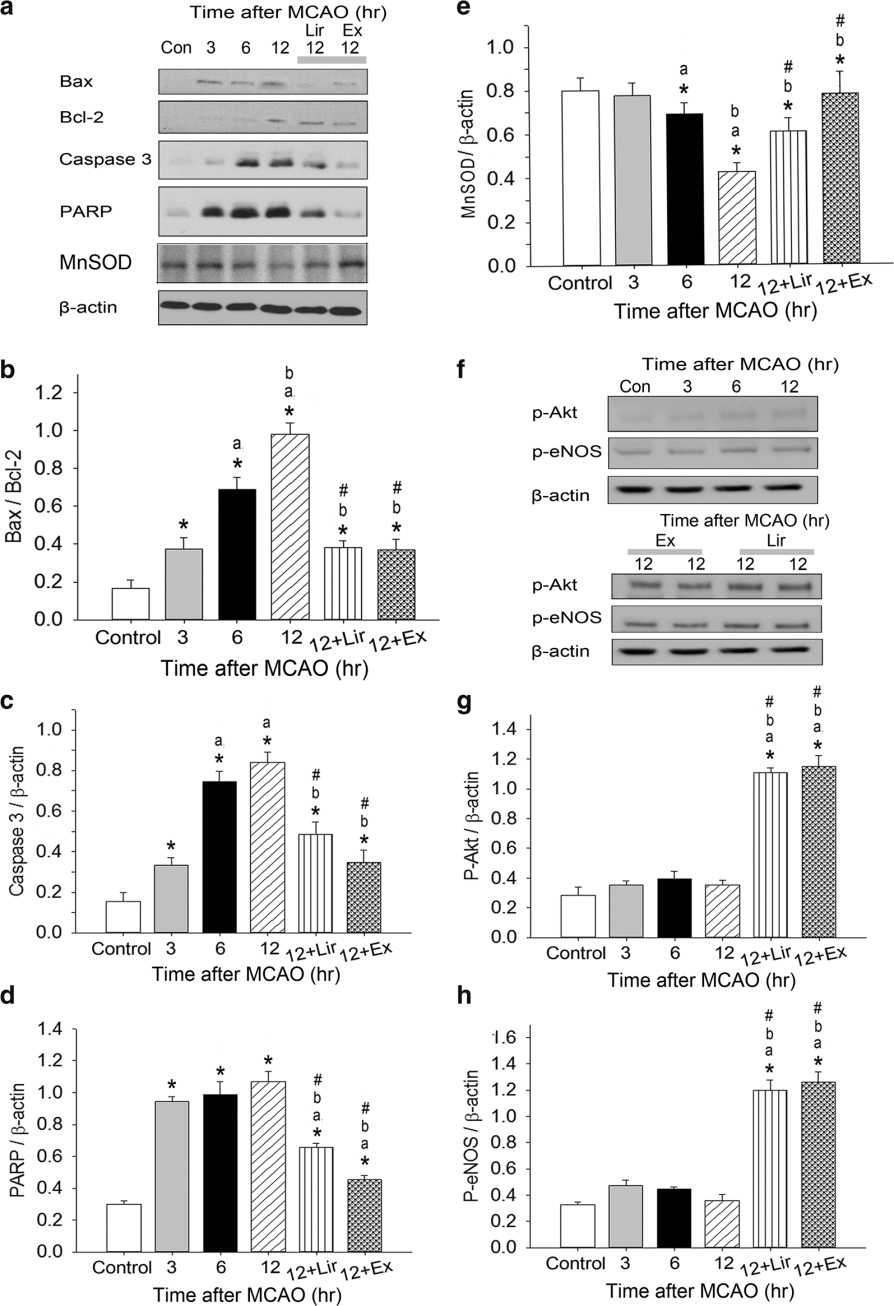
Effects of exendin-4 and liraglutide on MCAO-induced apoptosis-related protein expression. Typical western blots of Bax, B-cell lymphoma 2 (Bcl-2), caspase 3, poly-(ADP-ribose)-polymerase (PARP), and manganese superoxide dismutase (MnSOD) expression **a** and **f** phosphorylated Akt (p-Akt) and endothelial nitric oxide synthase (p-eNOS) levels **b** in the right cerebral cortex of sham-operated control or MCAO-injured db/db mice at 3, 6, or 12 h after surgery. Densitometric quantification of the ratio of Bax/Bcl-2 bands (**b**) and caspase 3 (**c**), PARP (**d**), MnSOD (**e**), p-Akt (**g**), and p-eNOS (**h**) bands normalized to β-actin. Data are presented as mean ± SEM (n = 6 per condition). Groups were compared using two-way ANOVA with a post hoc Bonferroni adjustment for pairwise comparisons. *P < 0.05 compared to control; ^a^ *P* < 0.05 compared to 3 h after MCAO; ^b^ *P* < 0.05 compared to 6 h after MCAO; ^#^ *P* < 0.05 compared to 12 h after MCAO

Figure 5a shows representative western blots of the apoptosis-related proteins Bax, B-cell lymphoma-2 (Bcl-2), caspase 3, and poly-(ADP-ribose)-polymerase (PARP) in the cortex of db/db mice after MCAO. The ratio of Bax to Bcl-2 expression (Fig. 5b), as well as the levels of caspase 3 (Fig. 5c) and PARP (Fig. 5d), were significantly higher than in control mice at 3, 6, and 12 h after MCAO (*P* < 0.05). These increases were significantly attenuated by Ex-4 and Lir treatments 12 h after MCAO (*P* < 0.05). The expression of caspase 3 and PARP showed non-significant decreases in mice treated with Ex-4 compared to those treated with Lir.

### Ex-4 and Lir treatments attenuate MCAO-induced decreases in manganese superoxide dismutase expression

The expression of the mitochondrial antioxidant enzyme manganese superoxide dismutase (MnSOD) was significantly reduced at 6 or 12 h after MCAO injury when compared to control mice (Fig. 5e); this effect was significantly attenuated at 12 h after MCAO by Lir and Ex-4 treatment (*P* < 0.05). Although Ex-4 resulted in a larger increase in MnSOD expression than did Lir, this difference was not significant.

### Ex-4 and Lir enhance levels of phosphorylated Akt and endothelial nitric oxide synthase in the brain

Figure 5f shows representative western blots of phosphorylated Akt (p-Akt) and endothelial nitric oxide synthase (p-eNOS) in the cortex of db/db mice after MCAO. Although neither p-Akt (Fig. 5g) nor p-eNOS (Fig. 5h) expression was affected by MCAO, the expression of both proteins was significantly increased in mice treated with Ex-4 or Lir 12 h after MCAO (*P* < 0.05). The expression of p-Akt and p-eNOS was similar in Ex-4- and Lir-treated animals.

### Ex-4 treatment ameliorates MCAO-induced motor and cognitive deficits

**Fig. 6:**
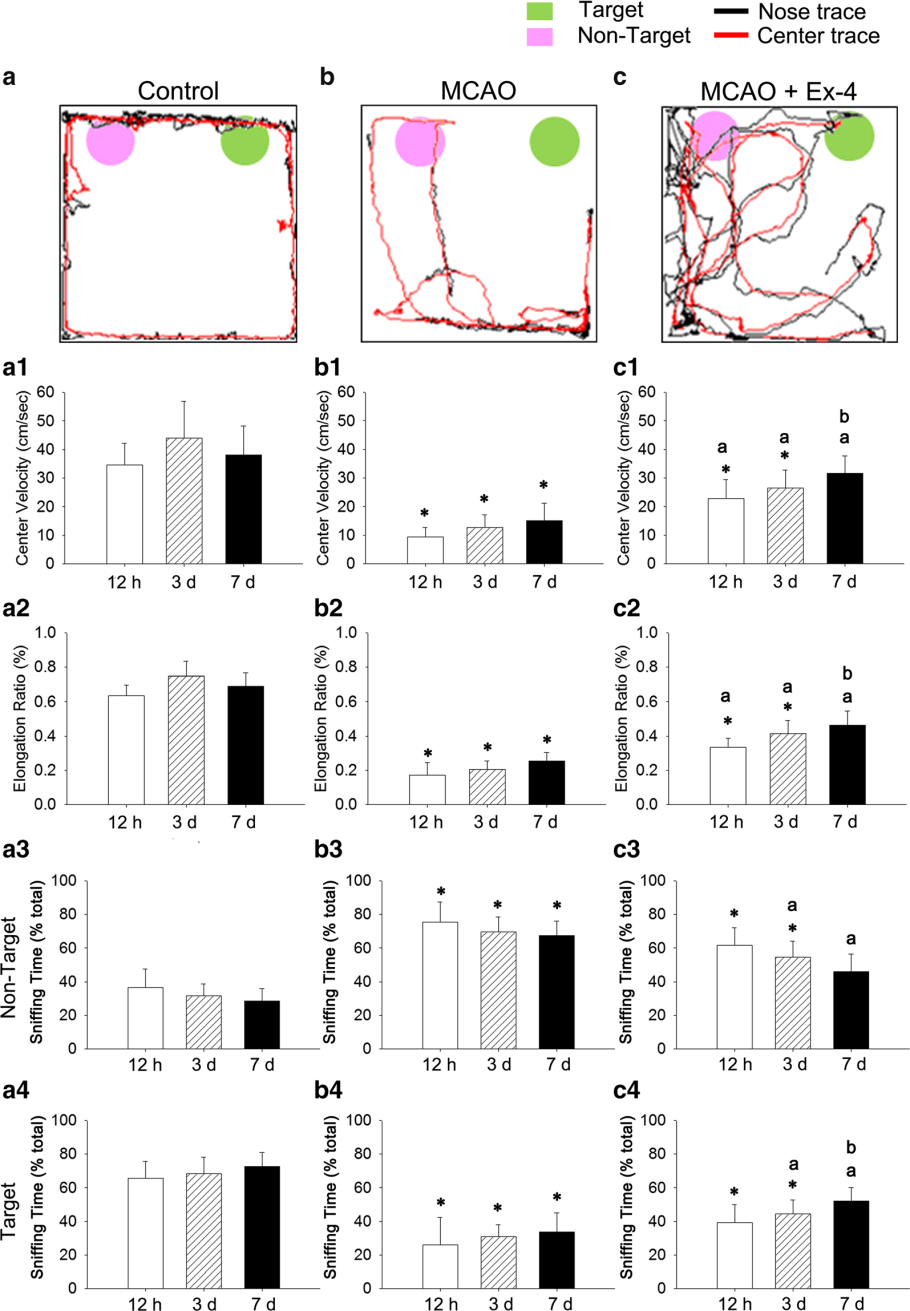
Effects of exendin-4 on MCAO-induced motor and cognitive deficits in db/db mice at 12 h (h), 3 and 7 days (d) after MCAO. **a**, **a1**–**a4** Experimental design and representative activity traces of control, **b**, **b1**–**b4** MCAO-injured, and **c**, **c1**–**c4** MCAO-injured mice treated with Ex-4 at 12 h, 3 days, and 7 days after injury. Motor parameters were (**a1**, **b1**, **c1**) velocity calculated using the body center trace and (**a2**, **b2**, **c2**) elongation ratio; cognitive parameters were (**a3**, **b3**, **c3**) non-target and (**a4**, **b4**, **c4**) target sniffing bousts. Groups were compared using two-way ANOVA with a post hoc Bonferroni adjustment for pairwise comparisons. *P < 0.05 compared to the time-matched control group; ^a^ *P* < 0.05 compared to the time-matched MCAO group; ^b^ *P* < 0.05 compared to MCAO + Ex-4 group at 12 h after injury

The effects of MCAO on motor and cognitive function were evaluated using a sniffing behavior paradigm. MCAO caused motor deficits, as shown by significant decreases in the average center velocity (Fig. 6b, b1) and elongation ratio (Fig. 6b2) at 12 h, 3 days, or 7 days after MCAO injury when compared to the time-match of control group (Fig. 6a–a2). Ex-4 treatment significantly ameliorated these MCAO-induced motor deficits at each time point. Further, the MCAO-induced increase in center velocity (Fig. 6c, c1) and elongation ratio were significantly improved by Ex-4 at 7 days after MCAO compared to 12 h after MCAO (Fig. 6c, c2) (*P* < 0.05).

Cognitive function was also significantly impaired by MCAO (Fig. 6b3, b4), as indicated by an increased percentage of non-target sniff time (Fig. 6c3) and decreased percentage of target sniff time (Fig. 6c4) at 12 h, 3 days, and 7 days after MCAO injury. Ex-4 treatment partially attenuated this cognition deficit, as the times spent investigating the non-target and target objects were significantly decreased and increased, respectively, at 3 and 7 days after MCAO in Ex-4-treated mice. We further found that the time spent exploring the target object was significantly increased at 7 days after MCAO compared to 12 h after MCAO in Ex-4-treated mice.

### Ex-4 and Lir treatments attenuate MCAO- induced voiding impairments

**Fig. 7:**
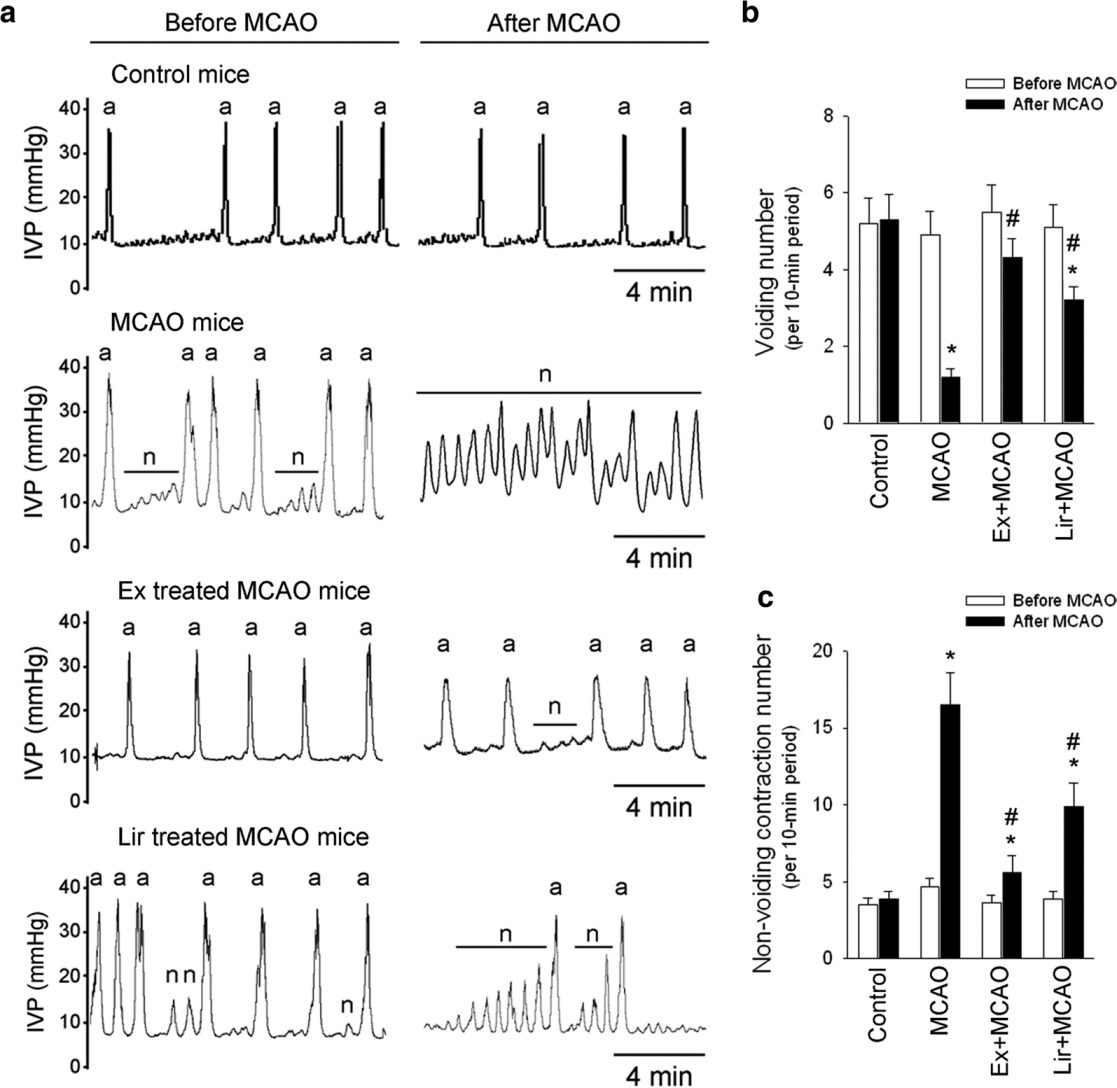
Effects of exendin-4 and liraglutide on MCAO-induced bladder dysfunction in db/db mice. **a** Typical intravesical pressure (IVP) traces before and after surgery in control, MCAO, Ex-4-treated MCAO, and Lir-treated MCAO groups. Voiding contractions (*a*) and nonvoiding contractions (*n*) are labeled. **b** MCAO significantly reduced voiding contractions and **c** increased nonvoiding contractions; these effects were partially reversed by Ex-4 and Lir treatments. Groups were compared using two-way ANOVA with a post hoc Bonferroni adjustment for pairwise comparisons. *P < 0.05 compared to the control group; ^#^ *P* < 0.05 compared to the MCAO group

Figure 7 shows the protective effects of Ex-4 and Lir against MCAO-induced bladder dysfunction in db/db mice. Figure 7a shows a representative recording of intravesical pressure (IVP) before and after MCAO injury in control, MCAO, Ex-4-treated MCAO, and Lir-treated MCAO groups. MCAO significantly reduced voiding contractions (Fig. 7b) and increased nonvoiding contractions (Fig. 7c); these effects were significantly attenuated by treatment with Ex-4 and Lir.

## Discussion

The results of this study demonstrate protective effects of the GLP-1 analogs Ex-4 and Lir against MCAO-induced cerebral blood flow impairment, blood and brain ROS production, oxidative stress-related and inflammatory protein expression, and impairments in motor and cognitive function and bladder contraction in the db/db mouse model of diabetes. MCAO decreased cerebral microcirculation in db/db mice and increased blood and brain ROS production and NF-κB, ICAM-1, caspase 3, and TUNEL expression. Ex-4 and Lir treatment in mice subjected to MCAO resulted in significantly higher cerebral blood flow, preservation of MnSOD expression, lower blood and brain ROS counts, and lower NF-κB, ICAM-1, caspase 3 and TUNEL expression compared to untreated MCAO mice. Expression of p-Akt and p-eNOS expression was not affected by MCAO but was significantly enhanced by Ex-4 and Lir treatments. Ex-4 and Lir treatments also mitigated MCAO-induced motor and cognitive deficits and micturition dysfunction, as indicated by reduced voiding contractions and increased nonvoiding contractions. As shown in the Fig. 1, magnetic resonance imaging data show that MCAO-induced brain edema was significantly reduced by Lir and Ex-4 treatments.

Several previous studies indicate that GLP-1 and GLP-1R agonists are neuroprotective [30, 31]. These molecules are small enough to cross the blood–brain barrier [32–34], and GLP-1Rs are widely expressed throughout the brain. Our recent data indicate that Ex-4 treatment reduces stroke-induced frontal cortex edema, endoplasmic reticulum stress, apoptosis, and upregulation of aquaporin 4, glial fibrillary acid protein, and ICAM-1 [3]. Briyal et al. [35] demonstrated that Ex-4 significantly protected rats from MCAO-induced cerebral ischemia, reducing the infarct volume by up to 27 % and reducing neurological deficits and oxidative stress parameters. These results suggest that Ex-4 and Lir are attractive candidates for reducing oxidative injury and inflammation in the brain. Elucidation of the molecular mechanisms of Ex-4 and Lir action in the brains of diabetic animals will require further investigation, although some reports have demonstrated that Ex-4 treatment in diabetic rats before and after stroke reduces ischemic brain damage by arresting microglial infiltration and increasing stroke-induced neural stem cell proliferation [36].

Brain ischemia and reperfusion initiate a complex cascade of metabolic events, several of which generate nitrogen and oxygen free radicals that mediate much of the damage that occurs after transient brain ischemia [37]. MCAO has previously been shown to increase the activity of NADPH oxidase and nitric oxide synthases in neurons, vascular endothelium, infiltrating neutrophils and macrophages, activated microglia, and astrocytes [3]; in the present study, we show that MCAO increases ICAM-1 expression and DHE labeling of ROS. We found that blood and brain ROS levels were significantly elevated after MCAO, especially at 12 h after injury. NF-κB nuclear translocation and ICAM-1 upregulation in the damaged brain cause leukocyte infiltration, adhesion, and migration, which lead to ROS production, inflammation, and oxidative injury. In this study, we observed that Ex-4 and Lir treatment significantly inhibited MCAO-induced NF-κB and ICAM-1 activation and ROS production. Teramoto et al. [34] demonstrated that suppression of oxidative damage is a key factor in neuroprotection. Although the exact mechanisms of Ex-4- and Lir-mediated reductions in MCAO-induced ROS production remain unclear, we suggest that Ex-4 and Lir act as antioxidants and anti-inflammatory agents to decrease MCAO-induced brain inflammation and injury.

Insulin and glucose levels can also influence brain injury. Evidence from several studies indicates that increased insulin can mitigate damage induced by ischemia and reperfusion. Hui et al. [38] showed that pretreating rats with insulin before cerebral ischemia significantly increases the number of surviving CA1 pyramidal cells in the hippocampus after 5 days of reperfusion. Another study reported that, in diabetic rats subjected to cerebral ischemia and reperfusion, insulin treatment decreased brain lesion volume, as well as basal and injury-induced apoptotis [39]. Our results show that Ex-4 exerts protective effects against ischemia-induced damage in db/db mice, which have higher serum insulin levels than normal mice. One previous study reported that Ex-4 treatment did not influence serum insulin levels in db/db mice [40]. Together, these results suggest that Ex-4 may be an appropriate therapeutic agent in diabetic stroke patients.

The effects of GLP-1 agonist treatment on the signaling pathways that affect oxidative stress- and apoptosis-related protein expression have been documented previously. Ex-4 directly improves impaired endothelium-dependent vasodilation of aortas via cAMP/AMPK-eNOS pathway [41]. Ex-4 also attenuates neointimal hyperplasia after vascular injury via camp/PKA pathway and inhibits TNFα production by peritoneal macrophages in response to inflammatory [25]. GLP-1 analog, Lir, promote neuronal survival and attenuate MDA oxidative stress and anti-apoptosis following by cerebral ischemia [35]. Ex-4 upregulates cAMP and triggers CREB phosphorylation [34], leading to the upregulation of Bcl-2 [42], which in turn inhibits apoptosis [43]. Our results show that Ex-4 and Lir significantly decrease Bax expression and the Bax/Bcl-2 ratio, leading to reduced apoptosis, possibly through stimulation of Akt and eNOS signaling. Akt pathway activation in ischemic regions has been reported to reduce ischemia- and reperfusion-induced damage by modulating the endothelial nitric oxide pathway [44]. In this study, we observed that Ex-4 and Lir treatments significantly increased p-Akt and p-eNOS levels following MCAO, indicating the recruitment of protective signaling pathways against oxidative stress.

Fu et al. [45] showed that cytokine and neuronal NOS levels are elevated in the lumbosacral spinal cord of rats subjected to MCAO, far from the injury site. In the spinal cord, tumor necrosis factor-α and interleukin-1β immunoreactivities were mainly localized to the ventral horn motor neurons contralateral to the site of MCAO. Neuronal NOS immunoreactivity and NADPH-diaphorase staining in the spinal cord were also markedly increased in response to MCAO. This, together with some neuronal deaths, may be linked to the dysfunction of the latter in a clinical stroke [45]. A previous study has demonstrated significant reductions in voided volume per micturition and significantly increased voiding frequency following mild forebrain ischemia in rats [14]. Consistent with these results, our unpublished data show that MCAO evokes frontal cortex injury in rats and inhibits efferent pelvic nerve activity, resulting in impaired voiding function, especially phase 2 of the voiding contraction, which is regulated by pelvic nerve activity. Ex-4 treatment restores pelvic parasympathetic nerve activity in these studies, indicating its protective effect against brain injury. As our data indicate that Ex-4 and Lir treatments ameliorate MCAO-induced bladder hyperactivity, we believe that Ex-4 or Lir may be promising therapeutic strategies for the treatment of stroke-induced bladder hyperactivity.

## Conclusion

Diabetic mice treated with GLP-1 agonists after ischemia/reperfusion injury (MCAO) exhibited increased cerebral blood flow, decreased ROS production, and decreased expression of oxidative stress-, inflammation-, and apoptosis-related proteins in the brain. Treatment with GLP-1 agonists also mitigated MCAO-evoked cognitive and motor impairments and stimulated protective signaling pathways and antioxidant MnSOD expression in the brain area that mediates parasympathetic/pelvic nerve-mediated voiding contractions. These results suggest that GLP-1 agonists may be useful in the treatment of cerebral and cardiovascular complications of diabetes.

## Methods

### Animals

The db/db mouse model of diabetes, which undergoes spontaneous, apoptotic destruction of pancreatic β-cells, was used in this study. Male db/db mice at 6–8 weeks of age were housed in a temperature-controlled facility at the Experimental Animal Center at I-Shou University (Kaohsiung, Taiwan) with a 12 h light/dark cycle. Animal care and experimental protocols were in accordance with guidelines prescribed by the National Science Council of the Republic of China (NSC1997). The animal studies were reviewed and approved by the Institutional Animal Care and Use Committee of I-Shou University (IACUC Protocol No. AUP-95-55-001 and Approval No. IACUC-ISU-95004).

### Middle cerebral artery occlusion

Transient MCAO using an intraluminal suture was performed as previously described [46]. Mice were anesthetized with chloral hydrate (400 mg/kg) in 25 % O_2_-enriched room air with spontaneous ventilation. Animals were placed in the supine position, and a midline incision was made along the neck. Using a surgical microscope, a silicone-coated 7.0 nylon filament (0.24 mm in diameter) was inserted through a small incision in the external carotid artery and advanced approximately 7 mm through the internal carotid artery towards the Circle of Willis and to the point of origin of the middle cerebral artery. After 60 min, the filament was removed to restore blood flow to the brain, and reperfusion was allowed for 0, 3, 6, or 12 h (n = 6 animals per group). Body temperature was maintained within the physiological range during MCAO and reperfusion.

### Cerebral cortex microcirculation measurements

Ex-4 and Lir (0.1 mg/mL) were administered intraperitoneally during the 0, 3, 6, or 12 h reperfusion periods following MCAO. Mice in the control group (0 h of treatment; n = 6) were treated with normal saline. We continuously measured cerebral cortex microcirculation in the control and DM mice during administration of vehicle, Ex-4, or Lir 3 or 6 h before or 12 h after MCAO as described previously [3]. A full field laser perfusion imaging system (moorFLPI; Moor Instruments, Wilmington, DE, USA) was used to continuously monitor microcirculation. Microcirculation in each region of interest was recorded in perfusion units, an arbitrary unit related to the product of average speed and the concentration of moving red blood cells in the tissue sample volume. The images were analyzed in real time using moorFLPI software version 3.0 (Moor Instruments Ltd.).

### Cerebral edema measurement by T2-weighted magnetic resonance imaging (MRI)

MRI was carried out in the animals using a Bruker Biospec 7-T MRI system as described previously [3]. Ex-4 and Lir (0.1 mg/mL) were administered intraperitoneally 12 h reperfusion periods following MCAO. Mice in the control group (0 h of treatment; n = 6) were treated with normal saline. Anesthesia was induced with 5 % halothane and maintained with 1.5 % halothane (both concentrations prepared in O_2_:N_2_O, 70:30 by volume). Control or DM rats treated with vehicle, Ex-4 or Lir post 12-h CAO were intubated and mechanically ventilated at a rate of 60 breaths/min. Heart rates and respiratory rates were monitored throughout the procedure, and body temperature was maintained at 37 °C. A rapid-acquisition relaxation enhancement T2-weighted sequence was used to determine the precise lesion location, with a rapid-acquisition relaxation enhancement factor (RARE) of 16, a repetition time of 5086 ms, and an echo time of 70.1 ms. The in-plane resolution was 250 × 250 × 250 µm and 15 slices were acquired. A second T2-weighted image set of 25 contiguous slices was acquired at the lesion site (RARE factor = 16, repetition time = 5086 ms, echo time = 70.1 ms) with an in-plane resolution of 117 × 117 × 500 µm. Infarct areas, outline in white, were calculated from each MRI image using image analysis software (Adobe Photoshop CS4). The percentage of edema was calculated as: Brain edema (%) = Infarct area/(Infarct area and unaffected area) × 100 %.

### Motor and cognitive function assessment

Motor and cognitive function were evaluated in Ex-4- or Lir-treated db/db mice using a novel sniffing behavior test before MCAO and 12 h, 3 days, or 7 days after MCAO. In the conditioning session, the animals were placed individually in a conditioning chamber (60 × 60 × 60 cm) for 2 h. Each chamber contained 2 objects: sawdust (nontarget object) and feed (target object). For the test session, the animal was placed in the same chamber for 15 min, and activity was recorded using a high-throughput-screening top-view camera (STD-CA67D-IR; Sony, Tokyo, Japan) and ObjectScan behavior analysis software (Clever Sys Inc., Reston, VA, USA) [3, 47]. Time spent exploring each object was recorded to assess cognitive behavior. All nose entries within 2 cm of the object were recorded as time exploring the object. Animals that spent <7 min exploring the objects during the test session were excluded from the analysis. Nose and center traces were recorded, and velocity and elongation ratio were analyzed to evaluate motor function. The objects and arenas were thoroughly cleaned with 70 % isopropanol between trials.

### Cystometry

Mice were anesthetized via subcutaneous injection of urethane (1.2 g/kg). An incision was then made in the caudal aspect of the abdomen to expose the bladder, a suture was placed in the dome of the bladder using 6-0 silk, and a PE-50 bladder catheter was inserted through the apex of the bladder dome and connected via a T-tube to an infusion pump and P23 ID pressure transducer (Gould Instruments, Cleveland, OH, USA), as described previously [48]. IVP was recorded continuously using a PowerLab 16S system (ADInstruments, Sydney, Australia). Sterile saline (0.9 %) was infused into the bladder at a rate of 0.8 mL/h. Mice were placed in a ventral recumbent position on a heating pad, and IVP was continuously recorded for at least 90 min for each mouse. Data were analyzed from traces of at least 5 consecutive voiding episodes obtained after a consistent voiding pattern had been achieved. Parameters measured included the intercontractile interval (defined as the time between peaks in IVP) and the maximal IVP associated with each voiding episode. Contractions with a contractile amplitude lower than the maximal IVP without actual voiding were designated as nonvoiding contractions. Mice were euthanized by an intraperitoneal injection of pentobarbital sodium (75 mg/kg) at the conclusion of data collection.

### Measurement of ROS production, NF-κB, ICAM-1 and apoptosis

Mice were sacrificed at the end of the experiment, and we conducted DHE 2,7-diamino-10-ethyl-9-phenyl-9,10-dihydrophenanthridine) and ICAM-1 staining to assess ROS production in the brain after MCAO. Brain tissue samples were frozen in Tissue-Tek optimal cutting temperature compound (Sakura Finetek, Torrance, CA, USA) and cut into 10-mm cross sections. To assess oxidative stress and apoptosis, fluorescent DHE and ICAM-1 stains and colorimetric caspase 3 and TUNEL stains were applied to each cross section for 5 min at room temperature. Images from brain stem sections were captured using a Leica DMRD fluorescence microscope (Leica Microsystems, Wetzlar, Germany) [49]. The brain tissue for assessment of NF-κB expression were washed with phosphate-buffered saline, stained with NF-κB polyclonal antibody (R&D Systems, Minneapolis, MN, USA) and mounted in mounting medium (Lecia). The slides were scanned under a Lecia TCS SP3 laser confocal microscope to obtain the confocal images.

As an additional measure of ROS levels in brain tissue, 1 mg samples were obtained after MCAO and homogenized in 0.2 mL of saline. Tissue ROS levels were immediately measured using the lucigenin-enhanced chemiluminescence method, which has been used previously to measure increases in ROS production in response to neurogenic inflammation [49]. A 100-s background luminescence measurement was recorded using a chemiluminescence analyzing system. Lucigenin solution (0.1 mM in phosphate-buffered saline, pH 7.4; 0.5 mL) was then added to the sample, and chemiluminescence was measured for 10 min. The area under the chemiluminescence curve was integrated to obtain the total chemiluminescence signal, and the background luminescence was subtracted. Duplicate tests were performed for each sample. Results are expressed as chemiluminescence counts per 10 s. A similar technique was used to measure blood ROS production, as described previously [48]. Lipid peroxidation was measured in cortical samples using an MDA colorimetric/fluorometric assay kit (Biovision, Milpitas, CA, USA).

### Western blotting

Western blotting was used to measure cortical levels of the apoptosis-related proteins Bax, Bcl-2, caspase 3, PARP, e-NOS, NF-κB, ICAM-1 and MnSOD. In brief, whole cortical lysate proteins (20 μg/well) were electrophoretically separated on 10 % polyacrylamide gels and transferred to nitrocellulose membranes (GE Healthcare, Westborough, MA, USA). The membranes were blocked and then incubated overnight at 4 °C with antibodies raised against the following proteins: Bax (AB2916; EMD Millipore, Billerica, MA, USA), Bcl-2 (Transduction, Bluegrass-Lexington, KY, USA), the 17 kDa activated cleavage product of caspase 3 (CPP32/Yama/Apopain; human full-length caspase 3 fusion protein containing a histidine-6 tag), the p85 N-terminal cleavage product of PARP (Promega, Madison, WI, USA); eNOS (BD Biosciences, San Jose, CA, USA), NF-κB (R&D Systems, Minneapolis, MN, USA), ICAM-1 (R&D Systems), and MnSOD (Enzo Life Sciences, Farmingdale, NY, USA). Antibodies were diluted 200- to 2000-fold. Membranes were then incubated for 1 h at room temperature with anti-rabbit or rabbit anti-sheep IgG antibodies conjugated to horseradish peroxidase (Vector Laboratories, Burlingame, CA, USA), and proteins were visualized using a commercial enhanced chemiluminescence kit (GE Healthcare). Band densities were determined semi-quantitatively by densitometry using an Alpha Innotech image analysis system (San Leandro, CA, USA) [50].

### Statistical analysis

Data are presented as mean ± standard error of the mean. The 2-sample t test was used to compare data between 2 groups. Two-way analyses of variance with post hoc Bonferroni correction for pairwise comparisons were used to analyze differences between 3 groups. All statistical analyses were 2-tailed and considered significant if *P* < 0.05. Statistical analyses were performed using SPSS v. 18.0 statistical analysis software (SPSS Inc., Chicago, IL, USA).

## Abbreviations

Bcl-2: B cell lymphoma-2
DHE: dihydroethidium (2,7-diamino-10-ethyl-9-phenyl-9,10-dihydrophenanthridine)
eNOS: endothelial nitric oxide synthase
Ex-4: exendin-4
GLP-1: glucagon-like peptide-1
ICAM-1: intercellular adhesion molecule-1
IVP: intravesical pressure
Lir: liraglutide
MCAO: middle cerebral artery occlusion
MDA: malondialdehyde
MnSOD: manganese superoxide dismutase
NADPH: nicotinamide adenine dinucleotide phosphate
NF-κB: nuclear factor kappa B
p-Akt: phosphorylated Akt (protein kinase B)
PARP: poly-(ADP-ribose)-polymerase
PU: perfusion units
ROS: reactive oxygen spec
